# Nutritional Characterization of Whole Mangosteen Pulp with Seeds and Its Application as an Alternate Functional Ingredient in Crackers

**DOI:** 10.3390/foods13182987

**Published:** 2024-09-20

**Authors:** Nisa Saelee, Roberto Castro-Muñoz, Worawan Panpipat, Manat Chaijan

**Affiliations:** 1 School of Agricultural Technology and Food Industry, Walailak University, Nakhon Si Thammarat 80160, Thailand; pworawan@wu.ac.th (W.P.); cmanat@wu.ac.th (M.C.); 2 Department of Sanitary Engineering, Faculty of Civil and Environmental Engineering, Gdańsk University of Technology, G. Narutowicza St. 11/12, 80-233 Gdansk, Poland; food.biotechnology88@gmail.com; 3 Food Technology and Innovation Research Center of Excellence, Walailak University, Nakhon Si Thammarat 80160, Thailand

**Keywords:** mangosteen, mangosteen seed, functional ingredient, functional crackers, mangosteen-based cracker

## Abstract

Mangosteen (*Garcinia mangostana* L.) fruits are high in nutrients and phytochemical compounds. The use of fresh whole mangosteen fruit pulp, including the seeds (MFS), instead of flour and sugar in crackers not only enhances the functional nutritional and medicinal benefits for consumers but also adds value to the products. The study investigated the nutritional value of MFS and then employed MFS to formulate MFS-based crackers with varying levels of MFS substitution in order to develop crackers enriched with functional ingredients. Proximate compositions, amino acids, sugars, minerals, fatty acids, color, texture, and antiradical properties were analyzed in fresh MFS and MFS-based crackers. The results indicated that MFS can be a source of crude fiber, minerals, amino acids, omega-6, and omega-9 fatty acids. Adding 13%, 18%, and 23% ground MFS to the crackers improved their nutritional value and physical characteristics compared to the control (0% MFS). MFS-based crackers promoted significantly (*p* < 0.05) higher fiber (4.04 ± 0.00–5.66 ± 0.01%gdw), ash (2.45 ± 0.00–2.74 ± 0.01%gdw), and protein (4.72 ± 0.00–7.72 ± 0.05%gdw) than the control without MFS addition. Carbohydrates (including dietary fiber) and total sugar decreased significantly (*p* < 0.05) to 57.68 ± 0.00–55.21 ± 0.11%gdw and 2.37 ± 0.00–4.42 ± 0.01%gdw, respectively, in all MFS-based crackers compared to the control basal cracker with added sugar. Moreover, MFS-based crackers contained oleic acid (C18:1, omega-9) at 5.19–5.78%gdw and linoleic acid (C18:2, omega-6) at 0.63–0.77%gdw. Furthermore, the MFS-based crackers had higher levels of minerals (i.e., potassium, phosphorus, sulfur, calcium, and magnesium) and bioactive compounds such as total phenolic acid and total flavonoid, as well as antiradical activity. This study revealed that MFS can be applied as an alternative functional ingredient in the manufacturing of nutritious cracker products, and the findings could potentially be implemented to promote the utilization of mangosteen seed as a sustainable agricultural product and waste-reducing method.

## 1. Introduction

Increasing demand in the health and functional food fields has led to an increased interest in the production of functional foods enriched with functional ingredients that can offer health benefits or prevent disease at safe concentrations and the intended benefit. This is a more appealing option for enhancing health through everyday meals compared to traditional dietary supplements [1,2,3]. Functional food ingredients are certain substances, either natural or synthetic, that are believed to offer health benefits beyond their basic nutritional value. These substances include bioactive compounds, lipids, vitamins, minerals, proteins and amino acids, carbohydrates, probiotics and prebiotics, and phytochemicals. They can be utilized in various products such as food, beverages, nutraceuticals, and dietary supplements [4,5,6]. Crackers are popular snacks consumed by individuals of all age groups. They are available in different flavors, shapes, and sizes and are made using wheat flour or mixed with other ingredients. They are shaped, baked, and packed in sealed containers. The global biscuits market reached a size of US$ 123.2 billion in 2023 with an expected market growth rate of 4.4% during 2024–2032 [7]. In 2024, the sales volume of crackers and biscuits in Thailand reached approximately 8.86 thousand metric tons [8]. Crackers can be made from a variety of flours and named according to their ingredients and spreading methods. They can be healthy snacks [9]. A variety of ingredients were used to produce crackers which were claimed as healthy products. These encompassed crackers made with dehulled oat flour and pea protein isolate [10], crackers based on brown rice [11], crackers enhanced with camelina oil [1], oyster mushroom crackers based on pearl millet [12], crackers made with rice flour, herbs, and spices [13], crackers made with cottonseed flour and enzyme addition [14], crackers based on *Macrotermes subhumans* flour [15], leftover rice crackers [16], and a gluten-free biscuit made with okara and jackfruit seed flour [17]. In general, the main ingredients of basic crackers are wheat flour, sugar, and fat. Excessive consumption of crackers can lead to health issues [17]. Efforts to promote the value-added, nutritional quality, and health benefits of crackers include the use of novel functional ingredients, nutraceutical active components, protein, minerals, pigment enhancements, bioactive compounds (particularly phenolic compounds with antioxidant capacity), and reduced sugar or carbohydrate content in cracker products [12,13,16,17]. The formulations created by adding functional ingredients and bioactive compounds were linked to human health benefits [10].

Mangosteen, also known as purple mangosteen (*Garcinia mangostana* L.), is a tropical fruit that thrives in Southeast Asia and tropical regions of South America. Thailand stands out as one of the leading mangosteen-producing countries in the world, with significant potential for exports. In 2023, the production volume of mangosteens in Thailand was 148.36 thousand metric tons. The global mangosteen market reached US$ 326 million in 2022 and is projected to reach US$ 658 million by 2030, with an expected market growth of 3.6% during the period of 2023–2030 [18,19,20]. When ripe, the mangosteen fruit displays a red-purple to dark purple color. The fruit’s characteristics may vary depending on the environment in which it is grown. The edible part, known as the white pulp or endocarp, makes up 20.6–30% of the fruit and is white, juicy, soft, and fluffy with a mildly sweet and sour taste. Additionally, it has a sweet and slightly sour aroma. The non-edible peel or pericarp constitutes 62.2% of the fruit, and the portion of mangosteen juice contained is 10.88% [18,21]. The flavor, color, and aroma of mangosteen comes from the metabolites such as *L*-mannopyranose, myo-inositol, arabinofuranose, galacturonic acid, *L*-(+)-tartaric acid, aspartic acid, neoisostegane, epirobinetinidol-(4β,8)-catechin, α-mangostin, and gartanin [22].

The mangosteen fruit is a valuable choice as a functional and beneficial healthy food because it has numerous biological and pharmacological activities. These include antioxidant, anti-inflammatory, antibacterial, antifungal, antimalarial, antidiabetic, and anticancer properties [23,24]. Furthermore, various metabolites such as sugars and derivatives, amino acids and derivatives, organic acids, alcohol, aldehydes, glycosides, fatty acids, phenolics, alkaloids, terpenoids, xanthones, and a quinone have been found in different stages of ripening [22]. In Thailand, mangosteen pulp is used in a variety of food products such as jam, juice, and sauce, while mangosteen peel can be used to produce extract incorporated into pharmaceutical products, cosmetics, and personalized personal care items such as cream, bar soap, lip balm, lotion, toner, and toothpaste [25]. The seeds of the mangosteen contain beneficial nutrients such as protein, carbohydrates, minerals, fats, and fatty acids, which have high bioactive properties. Some of these bioactive compounds, such as alpha-mangostin, exhibit antioxidant activity, leading to stronger DPPH inhibitory power values. Antioxidants, including phenolic acids, flavonoids, terpenes, tocopherols, vitamin C (ascorbic acid), and carotenoids, have high TPC values [26,27]. Proximate analysis indicated that the seeds contained a high amount of carbohydrates and were rich in oil (21.68 ± 6.18%), but had a low protein content [28].

Mangosteen seeds might be edible or inedible, based on the customary eating practices of the consumer. When pulp-based products are being produced, seed may be regarded as waste. Reports state they have minimal to no toxicity and high nutritional value, making them potential raw materials for food and pharmaceutical products [26]. On the other hand, when consumed fresh, some individuals enjoy the entire flesh, including the seed. The potential for upcycling these materials as food ingredients is significant due to their diverse array of nutrients, along with their capacity to deliver therapeutic and functional benefits. Because of the healthy components in mangosteen seeds, employing whole pulp with seed may therefore be advantageous for both human health and the fruit processing sector because it minimizes waste. The seed is not consumed or utilized for any industrial purposes. Prior to using the whole mangosteen pulp with seed as a functional ingredient in food preparation, the nutritional content should be ascertained. The objective of this research was to evaluate the nutritional value of the whole mangosteen fruit pulp with seed (MFS) and the use of MFS as an alternate functional ingredient in crackers. The study focused on analyzing the physicochemical characteristics, bioactive properties, fatty acid changes, and nutritional value of MFS-based crackers as a novel, alternative functional snack.

## 2. Materials and Methods

### 2.1. Sample Preparation

The samples of mangosteen fruit were obtained commercially from a local farm in Nakhon Si Thammarat, Thailand. According to the LC-MS/MS analysis with the in-house method, STM No. 03-186, based on EN 15662:2018 [29], mangosteen fruits were devoid of 4 types of pesticide contaminants: organophosphate, organochlorine, carbamate, and pyrethroid. Fruits were stored at room temperature (27–29 °C) until their color turned dark purple at stage 5 of ripening. The skin was then peeled. The flesh of the whole mangosteen fruit including the seed (MFS) was collected and stored at −1 °C for no more than a month before being employed in experiments. The frozen MFS was then thawed and ground at 4 °C with a kitchen grinder at 21,000 rpm for 1 min (Ultramax Plus, Bangkok, Thailand) until it was fine, before being tested and used for mangosteen-based cracker production.

### 2.2. Preparation of MFS-Based Crackers

**Figure 1:**
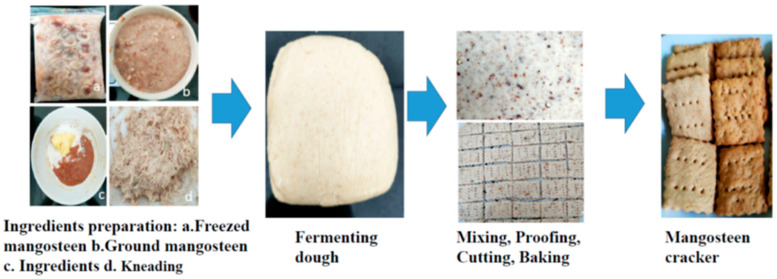
The production process of the whole mangosteen fruit pulp including seed (MFS)-based crackers.

The impact of MFS addition on the physicochemical properties of the crackers was investigated. The dough for the crackers was made up of 125 g of all-purpose wheat flour (Frog, Thaiflourmill, Samut Prakan, Thailand), 38 g of white sugar (Lin, Thairoongruang sugar Group, Uthai Thani, Thailand), 55 g of fresh milk (Meiji, CP-Meiji, Saraburi, Thailand), 65 g of unsalted butter (Home Fresh Butter Gold, Century House Dairy Co., Ltd., Ratchaburi, Thailand), 2 g of dry yeast (Saf-instant, Saf Yeast Co., Pvt. Ltd., Uttar Pradesh, India), 3 g of baking soda (Mc Garrett, Kosher Thaikashrut, Bangkok, Thailand), and 2 g of salt (Prung Thip, Thai Refined Salt Co., Ltd., Bangkok, Thailand). Four different formulas with varying ratios of MFS at 0%, 13%, 18%, and 23% (*w*/*w*) were tested. Sugars and flour were replaced with MFS as specified in Table 1. The mixtures were kneaded, wrapped in polyethylene bags, and left to form dough at room temperature for 2 h. The fermented dough was then manually rolled out into a thin sheet of 3 mm thickness and cut into the cracker sheets. Baking was carried out in a baking oven (Cuizimate, Bangkok, Thailand) at 200 °C for 15 min (Figure 1). After cooling to room temperature, the crackers were stored in a sealed plastic container for further analysis.

### 2.3. Proximate Analysis

A proximate analysis was conducted on the MFS and MFS-based crackers containing 0%, 13%, 18%, and 23% MFS. The following analyses were performed: crude fiber and dietary fiber contents (AOAC No. 985.29), crude fat content (STM No. 03-184 and AOAC No. 2008.06), crude protein content (AOAC No. 981.10), ash content (AOAC No. 945.38C), and moisture content (AOAC No. 945.38B), in accordance with the recommendations of the Association of Official Analytical Chemists [29]. Total carbohydrate analysis and calories were determined according to the Method of Analysis for Nutrition Labeling, 1993, p. 106 [30]. Carbohydrate content was calculated using the equation: carbohydrate (%) = 100 − %moisture − %fat − %fiber − %protein − %ash.

### 2.4. Amino Acid, Sugar, Mineral, and Fatty Acid Analysis

The amino acid profile of MFS was analyzed using AOAC [18] method 994.12.

Analysis of various sugars (glucose, fructose, sucrose, maltose, and lactose) in the MFS and MFS-based crackers involved diluting the sample, clarifying it via centrifugation at 10,000 rpm for 10 min at 4 °C, filtering through 0.45 µm, and analyzing it using HPLC with Aminex HPX-87P (Biorad, Hercules, CA, USA) using an RI detector and DI as mobile phase. Each type of sugar solution was used as an external standard, and the amount of each type of sugar was calculated from the retention time and area of the standard sugar. Total sugars were analyzed as the sum of tested sugars (glucose, fructose, sucrose, lactose, and maltose).

Important elements such as calcium, magnesium, zinc, potassium, phosphorus, boron, iron, nickel, manganese, copper, aluminum, and sodium of MFS and MFS-based crackers were analyzed using the Inductively Coupled Plasma Emission Spectroscopic Method (ICP-OES) with each standard solution as an external standard. The concentrations of each element were calculated based on AOAC No. 2011.19 [30]. The process involved taking more than 1.5 g of a finely ground sample, drying it at 100 °C overnight, then placing the dish in a 525 °C furnace to obtain ash. The ash was then dissolved in 1 M HNO_3_, adjusted, and diluted to the proper volume before analysis. The mineral concentrations were determined from their known calibration standard curve.

A gas chromatograph (Agilent, Santa Clara, CA, USA) equipped with a flame ionization detector (FID) was used to analyze the fatty acid profile of MFS and MFS-based crackers. One microliter of fatty acid methyl esters (FAME) was injected into an Rt-2560 GC Capillary Column, 100 m, 0.25 mm ID, 0.20 µm film thickness. The standard component FAME mix was initially analyzed according to AOAC method 996.06 [18]. Identification of fatty acids was conducted by comparing the retention times with the component FAME mix standards.

### 2.5. Determination of Color and Texture

Color value analysis was conducted using a Hunterlab Miniscan/EX instrument with 10° standard observers and a standard illuminant D65 (Hunter Assoc. Laboratory, Reston, VA, USA). Calibration with black and white standards was performed before directly measuring the color of the crackers. The color values were expressed as L* (lightness), a* (redness/greenness), and b* (yellowness/blueness).

Texture analysis was performed using a Texture Analyzer (LR5K, Lloyd Material Testing Instrument, Drive Largo, FL, USA) with NEXYGEN V.4 software data analysis and a 5 cm diameter flat cell. The conditions for analysis were as follows: 1 mm/s pretest speed, 5 mm/s test speed, 2 mm/s post speed, 5 N trigger force, and 5 cm distance. The hardness and the cohesiveness were recorded.

### 2.6. Determination of Total Phenolic Content (TPC), Total Flavonoid Content (TFC), and Antiradical Activity (ARA)

First, 15 g of ground sample (MFS and MFS-based crackers) was extracted with 75 mL of 80% methanol. The mixture was then shaken at 90 rpm for 24 h at room temperature. After extraction, the sample was centrifuged at 10,000× *g* for 10 min (RC-5B plus centrifuge, Sorvall, Norwalk, CT, USA). The resulting supernatant was collected and stored in a sealed container for further analysis.

For the TPC test, a 4 mL aliquot of the clear supernatant was mixed with 2.5 mL of 10% Folin–Ciocalteau’s reagent and 1.6 mL of 7% Na_2_CO_3_. The resulting mixtures were then incubated at room temperature for 90 min, and the absorbance was measured at 760 nm. Additionally, standard gallic acid solutions at various concentrations were prepared and analyzed using the same method. The obtained values were used to calculate the TPC value in mg gallic equivalent (mgGAE) per 100 g.

The TFC test was conducted by pipetting 1 mL of supernatant and adding 4 mL of distilled water, followed by the addition of 0.3 mL of 5% NaNO_2_, 0.3 mL of 10% AlCl_3_, and 2 mL of 1 M NaOH. The volume was then adjusted to 10 mL with distilled water, and the absorbance was measured at 510 nm. The absorbance values were compared with those obtained from standard catechin solutions at various concentrations to calculate the TFC value as the mg catechin equivalent (mg CCE)/100 g)

The ARA involved adding 0.1 mL of the supernatant into 5 mL of 0.1 mM 2,2-diphenyl-1-picrylhydrazylradical (DPPH) and then incubating the mixture in the dark at 27 °C for 13 min. The absorbance was measured at 517 nm, and the % ARA was calculated according to the method of Saelee [31].

### 2.7. Statistical Analysis

The entire investigation was conducted using a completely randomized design (CRD). All data are presented as mean ± standard deviation (S.D.) based on triplicate analysis. Statistical differences among the samples were analyzed using Minitab 16.0 software with analysis of variance (ANOVA) and one-way ANOVA. Grouping comparisons were made using the Tukey method, with a significance level set at *p* < 0.05.

## 3. Results and Discussion

### 3.1. Proximate Composition

The procedure for producing crackers containing MFS is outlined in Figure 1. Ground MFS was prepared according to the procedure specified in Section 2.2. Table 1 shows that the MFS-based crackers were manufactured by replacing 13, 18, and 23% ground MFS to wheat flour and reducing the sugar level to zero in comparison to the control without MFS (0% MFS).

The proximate compositions of MFS and MFS-based crackers are shown in Table 2. Moisture was the most abundant composition in MFS (78.83 ± 0.47% wet weight). The fresh MFS contained crude fiber, crude protein, crude fat, ash, and carbohydrate with average test values of 15.81 ± 0.17, 1.25 ± 0.00, 10.05 ± 0.11, 1.24 ± 0.01, and 90.95 ± 0.10 g/100 g dry weight (%gdw), respectively. The oil yield of the mangosteen seed is 21.18 ± 6.18 g/100 g, which is higher than MFS 10.05 ± 0.11 (%gdw). Mangosteen seeds can be effectively used as sources of dietary fiber due to their high crude fiber and carbohydrate contents [28].

When MFS was used to produce crackers, the total dietary fiber and total ash increased significantly (*p* < 0.05) to 4.04 ± 0.00, 4.34 ± 0.02, and 5.66 ± 0.01 and 2.45 ± 0.00, 2.6 ± 0.02, and 2.74 ± 0.01%gdw, respectively, when substituted with 13, 18, and 23% ground MFS. The original basal cracker formula comprised 5.43 ± 0.00%gdw of total dietary fiber and 2.22 ± 0.00%gdw of total ash. Both soluble and insoluble dietary fiber sources are commonly integrated into functional foods and have been shown to contribute to the reduction of LDL concentrations. Fruit pulp, a prevalent component, contains both forms of fiber, which are imperative for overall health, digestive processes, and disease prevention. [28]. Based on European dietary guidelines, it is recommended that adults maintain a daily intake of 25–35 g of dietary fiber. For men, the recommended intake is 30–35 g per day, while for women, it is 25–32 g per day [32]. Fresh MFS had a crude protein content of 1.25 ± 0.00%gdw, while MFS-based crackers had greater protein values but were lower than the basal formula (*p* < 0.05). MFS-based crackers had protein levels of 4.72 ± 0.00, 7.35 ± 0.01, and 7.72 ± 0.05%gdw at 13, 18, and 23% MFS substitution, respectively, which increased considerably at elevated MFS supplementation.

Compared to the control, which was sugar added to the basal cracker, carbohydrates (including dietary fiber) significantly decreased (*p* < 0.05) from 68.41 ± 0.04% at 0% MFS to 57.68 ± 0.00%, 57.48 ± 0.33%, and 55.21 ± 0.11%gdw at 13%, 18%, and 23% MFS substitution, respectively. MFS could be used to replace added sugar in cracker formulations because it contains a range of natural sugars (see Section 3.2). However, the addition of MFS to 23% resulted in a higher calorie content (559.39 ± 1.08 kcals/100 gdw) compared to 18% and 13% replacement, with values of 539.12 ± 3.13 and 541.48 ± 0.02 kcals/100 gdw, respectively. This could be related to the increased fat content in the finished crackers with higher MFS levels (*p* < 0.05). However, the plant-based oil may contain essential fatty acids that are beneficial for health (see fatty acid profile in Section 3.2)

### 3.2. Profiles of Amino Acid, Sugar, Mineral, and Fatty Acid

The amino acid content of fresh MFS is shown in Table 3. The most abundant amino acid discovered in MFS was tryptophan (0.19%), followed by alanine (0.10%), aspartic acid (0.08%), and glutamic acid (0.08%). Tryptophan, an amino acid, can reduce the time it takes to fall asleep [3]. Other amino acids identified included glycine, histidine, arginine, hydroxyproline, isoleucine, leucine, lysine, phenylalanine, proline, serine, threonine, tyrosine, methionine, and valine, while cystine and hydroxylysine were undetectable. Wheat flour is a primary ingredient in basal cracker formulation, although it contains low levels of essential amino acids such as lysine, methionine, and threonine. The essential amino acids in the crackers may be boosted, as a result of the presence of essential amino acids in MFS.

The sugar contents of fresh MFS, namely, sucrose, glucose, and fructose, were found to be 33.45 ± 0.37, 18.71 ± 0.21, and 19.94 ± 0.22%gdw, respectively. Sucrose was the main sugar found in MFS, while lactose and maltose could not be detected (Table 4).

The control cracker, without MFS, had a total sugar content of 20.79 ± 0.01%gdw. MFS-based crackers with no added sugar had a total sugar of 2.37 ± 0.00%, 3.58 ± 0.02%, and 4.42 ± 0.01% (dw) when flour was replaced at 13%, 18%, and 23%, respectively. The use of fresh MFS including mangosteen pulp and seed can minimize the amount of granulated sugar added to crackers, lowering processing costs. Sucrose content in MFS-based crackers ranged from 2.05 ± 0.00% to 3.87 ± 0.01% (dw), with fructose accounting for 0.31 ± 0.00% to 0.54 ± 0.00% (dw). Glucose was depleted during dough fermentation in MFS-based crackers (Table 4).

The most abundant element in fresh MFS was potassium (395.94 ± 8.73 mg per 100 g dry weight (%mgdw)), followed by magnesium (111.03 ± 2.45 %mgdw), phosphorous (68.98 ± 1.52 %mgdw), calcium (63.31 ± 1.40 %mgdw), sulfur (60.48 ± 1.33 %mgdw), and zinc (6.33 ± 0.14 %mgdw) (Table 5). The study conducted by Ajayi et al. [28] found that the seed flour had the highest levels of potassium (7071 mg/kg), magnesium (865 mg/kg), and calcium (454 mg/kg). Additionally, it contained trace amounts of manganese, iron, copper, sodium, and nickel. Manganese and copper function as co-factors for antioxidant enzymes, while potassium and calcium present potential as components in functional foods [3].

The substitution of MFS in cracker products at levels of 13, 18, and 23% significantly increased (*p* < 0.05) the quantity of potassium from 1213.05 ± 1.44 to 1437.68 ± 0.11, 1513.05 ± 17.51, and 1629.16 ± 6.27 mg per 100 g, respectively. For magnesium, it was increased from 165.18 ± 0.20 to 190.86 ± 0.01, 212.95 ± 2.46, and 232.44 ± 0.59 mg per 100 g. Calcium was increased from 434.51 ± 0.52 to 456.41 ± 0.03, 483.05 ± 5.59, and 523.51 ± 2.02 mg/kg. Additionally, trace elements such as copper were found to be 0.85 ± 0.00, 0.87 ± 0.00, 0.91 ± 0.01, and 0.95 ± 0.00 %mgdw, for samples added with 0, 13, 18, and 23% MFS, respectively. The addition of MFS led to higher levels of minerals in all test batches compared to crackers without MFS (Table 5). MFS-based crackers showed no difference in sodium content compared to the control, and the quantities of nickel and aluminum were found to be less than 0.2 and 1.0 mg/kg, respectively.

The addition of MFS resulted in greater amounts of most minerals, except iron and manganese, in all test batches compared to crackers without MFS, especially at high MFS levels (Table 5). Notably, MFS-based crackers were high in potassium, phosphorus, sulfur, calcium, and magnesium (Table 5).

According to Table 6, showing the fatty acid compositions, MFS contained several beneficial fatty acids. It included essential polyunsaturated fatty acids (PUFAs) such as linoleic acid (C18:2, omega-6) at 1.77%gdw and oleic acid (C18:1, omega-9) at 1.58%gdw, resulting in an unsaturated fat content of 3.35%gdw. Additionally, it contained saturated fatty acid at 6.00%gdw, with capric acid (C10:0) at 0.09%gdw, lauric acid (C12:0) at 0.05%gdw, palmitic acid (C16:0) at 0.60%gdw, stearic acid (C18:0) at 5.16%gdw, and arachidic acid (C20:0) at 0.05%gdw. The mangosteen seed oil extract contains a lower percentage of linoleic acid at 1.30% compared to the higher palmitic acid content of 49.5%, which was lower than in the mangosteen seed oil extract, which contained one essential fatty acid, linoleic acid, at 1.30%, but a lower amount of palmitic acid (49.5%) [28]. Mangosteen seed fat (MSF) extract is composed of high levels of stearic acid (57.9%), linoleic acid (20.3%), and oleic acid (16.0%) [33,34]. Ajayi et al. [32] highlighted that mangosteen seeds contain a high oil content (>20%) and could potentially serve as a valuable source of fat for industrial use. However, seeds are often ignored and underutilized, and only a few studies have investigated their fatty composition. Omega-3, omega-6, and omega-9 fatty acids are unsaturated fatty acids that offer health benefits and have various biological effects. In MFS, omega-9 is present in the form of oleic acid (C18:1, omega-9) at 1.58%gdw). Omega-9 polyunsaturated fats are considered partially essential fatty acids that provide a healthier alternative to saturated animal fats and have several health benefits, including anti-inflammatory and anti-cancer properties [35]. MFS-based crackers with 13%, 18%, and 23% flour replacement had oleic acid at 5.19, 5.31, and 5.78%gdw, respectively, which was greater than the control (0% MFS) at 3.60%gdw, possibly due to milk or butter in the cracker-based dough. Additionally, oleic acid has been used in the pharmaceutical industry as a solubilizing agent or emulsifier [35]. The MFS-based crackers contained omega-6 in the form of linoleic acid (C18:2, omega-6) at concentrations of 0.62, 0.63, 0.71, and 0.77%gdw with MFS substitutions of 0, 13, 18, and 23%, respectively. The amount of linoleic acid increased with the addition of MFS, reaching 0.63, 0.71, and 0.77%gdw at 13, 18, and 23% MFS substitution, respectively. Omega-6 fatty acids are essential for brain function and normal growth and development. As a type of PUFA, omega-6 helps stimulate skin and hair growth, maintain bone health, regulate metabolism, and support the reproductive system [35].

Omega-3 PUFAs were found in MFS-based crackers at levels of 0.18–0.26%gdw, while no detectable omega-3 was found in MFS. Generally, omega-3 is derived from plant sources in the form of linolenic acid (LNA, C18:3 omega-3), which is a precursor to eicosapentaenoic acid (EPA, C20:5 omega-3) and docosahexaenoic acid (DHA, C22:6 omega-3). These fatty acids have been shown to be beneficial and have exhibited positive effects in metabolic and cardiovascular disease prevention [36]. Additional fatty acid compositions, including PUFAs (arachidonic acid (C20:4, omega-6), eicosapentaenoic acid (C20:5, EPA, omega-3), and eicosatrienoic acid (C20:3, omega-6)), short chain saturated fatty acids (butyric acid (C4:0), caproic acid (C6:0) or hexanoic acid, and caprylic acid (C8:0) or octanoic acid), and other trace fatty acids were detected in the MFS-based crackers (Table 6).

### 3.3. Physical and Phytochemical Properties of MFS-Based Crackers

Color values are crucial in indicating the visual perception of taste, freshness, and quality of crackers. The L* values of cracker products with MFS tended to be lower than those without the addition (Table 7). This translates to a decrease in cracker brightness as wheat substitution levels increase. The a* value increased in crackers containing MFS compared to the control, resulting in a more yellow-brown appearance (Table 7). The b* value reduced, as evidenced by the decreased yellow shade in crackers containing MFS (Table 7). These changes were thought to be induced by the darkening of seeds caused by baking time and temperature, as well as non-enzymatic browning reactions mediated by the Maillard reaction, caramelization, and phenolic compounds oxidation in the final products [37]. Oppong et al. [38] reported that nonenzymatic browning reactions, particularly the Maillard reaction and caramelization, generally occurred upon heating of food containing reducing sugars and amino acids. Overall, color was mostly determined by the amount of flour used, as well as the type of flour, with higher degrees of substitution exerting a greater impact [39]. Furthermore, the complex chemical reaction in fresh MFS involving, for example, hemicellulose, tannin, and phenolic compounds, may also result in color changes in MFS-based crackers [40].

The texture of a cracker is an important factor that influences its quality and consumer acceptance. Texture is a complex property that encompasses various attributes. One such attribute is hardness, which refers to the amount of stress required to deform a food material to a certain level [25]. In the context of crackers, hardness is often associated with the level of difficulty in biting or the crispness of the product. Another important attribute is cohesiveness, which measures the degree to which a food can be deformed before it breaks, indicating the strength of internal bonds. High cohesiveness indicates a strong ability of the constituents to hold together [41].

In Table 7, the hardness and cohesiveness of MFS-based crackers varied among different formulas. The hardness values decreased significantly with the addition of MFS. However, with 13% MFS replacement, the cracker is tougher than the control and those at 18% and 23% MFS. This may be due to the high amount of flour in this formula and the low amount of MFS replacement. At 18% MFS, the cracker has a lower hardness of 5.74 ± 1.23 N than at 23% MFS, where it is 11.25 ± 0.68 N, which may be attributed to the higher moisture content (4.39 ± 1.12%) in the 18% MFS condition (see Table 2). Wang et al. [42] reported that crackers with lower moisture content are much easier to break than those with higher moisture content. Additionally, incorporating a small amount of pea fibers (5–10%) led to reduced elasticity in the dough. On the other hand, adding too much fiber (30%) resulted in a brittle, grainy texture that made the dough impossible to process [43]. This was possibly due to the limited internal gluten linkages, which means that even fiber could improve water and fat retention. Dietary fiber may have a positive impact on increasing the bread volume and crumb structure, while also limiting staling [44].

The addition of 13% MFS for flour substitution has significantly higher (*p* < 0.05) cohesiveness than the control and other conditions. There were no significant differences in the cohesiveness of the crackers with 18% and 23% MFS substitution compared to the control (*p* < 0.05). This means that the increased addition of MFS and constant wheat flour in the MFS-based formula could promote the cohesiveness of the resulting cracker. MFS may not interfere with the protein network, allowing it to withstand a greater degree of deformation before breaking [45]. In general, when water is present during mixing and kneading, gluten is formed from two proteins, glutenin and gliadin, which are found in wheat and grains. In MFS-based crackers, in addition to dietary fiber, some protein and sugars may help to adhere to specific ingredients, affecting the cohesiveness. However, Armstrong and Barringer [46] discovered that crackers with a high carbohydrate content, particularly sucrose, have been effectively used to adhere to powders and small particulates. Additionally, some hydrocolloids in MFS carbohydrates may interact with starch or gluten, which can affect the dough’s rheology and the quality of baking products. They have utilized hydrocolloids as fat replacers, gluten substitutes, and therapeutic fiber sources, intending to achieve a cracker with less gluten [47]. Wang et al. [8] indicated that cotton seed flour addition decreased the cracker breaking force and stack height due to the baking performance when producing high-protein content crackers.

When MFS was added to produce MFS-based cracker products, the levels of TPC, TFC, and ARA tended to increase when compared to the control. The highest TFC value of 92.2 ± 0.00 mg/100 g was observed when 23% of the ingredients was used. Interestingly, the highest TPC and ARA levels were noticeable in crackers with 13% MFS. Overall, the addition of mangosteen seeds enhanced the antioxidant activity of the products (Table 7). The antioxidant activity of MFS-based crackers was likely related to the ingredients utilized, as well as the Maillard reaction and caramelization products produced during baking [23]. TPC values in the mangosteen seed extract were also lower, ranging from 201.98 to 338.59 mgGAE/g extract [27]. Antioxidant defense activities lead to oxidative stress. Severe oxidative stress can result in mitochondrial and membrane mutations or disorders, as well as cell and tissue damage. Clemen-Pascual et al. [48] report the presence of phytochemicals such as flavonoids, glycosides, tannins, anthraquinones, saponins, steroids, and triterpenoids that exhibit astringent action, rapid healing, and the ability to form new tissues on wounds and inflamed mucosa.

## 4. Conclusions

The crude fiber, crude fat, ash, minerals, phenolic compounds, and amino acid content found in MFS make it a viable substitute functional ingredient for making crackers. The nutritious qualities of the resulting crackers were greatly enhanced by adding MFS, which was used to completely replace sugar and partially substitute flour. It should be highlighted that the MFS-based crackers had higher concentrations of omega-9 and omega-6 fatty acids than the control recipe. MFS-based crackers considerably increased the total phenolic content and antioxidant activity, which may have health benefits, especially at 13% substitution. Taking everything into consideration, this study offers foundational knowledge and references for the commercial usage of mangosteen in the future. However, additional studies should be done on MFS-based crackers’ sensory analysis and storage stability.

## Figures and Tables

**Table 1:** Formulation of the whole mangosteen fruit pulp including seed (MFS)-based crackers.

Ingredients (g)	0% MFS (Control)	13% MFS	18% MFS	23% MFS
Wheat flour	125	125	110	95
Sugar	38	0	0	0
MFS	0	38	53	68
Butter	65	65	65	65
Milk no fat	55	55	55	55
Salt	2	2	2	2
Yeast	2	2	2	2
Baking soda	3	3	3	3
Total	290	290	290	290

**Table 2:** Nutritional values of fresh whole mangosteen fruit pulp including seed (MFS) and MFS-based crackers made with various MFS levels.

MFS Substitution	Calories (Including Dietary Fiber) (kcals/100 gdw)	Carbohydrates (Including Dietary Fiber) (g/100 gdw)	Total Dietary Fiber (g/100 gdw)	Fat (g/100 gdw)	Protein (g/100 gdw)	Ash (g/100 gdw)	Moisture (%)
MFS	454.26 ± 4.97 d	90.95 ± 0.10 a	15.81 ± 0.17 a	10.05 ± 0.11 d	1.25 ± 0.00 e	1.24 ± 0.01 e	78.83 ± 0.47 a
0%	498.90 ± 0.30 c	68.41 ± 0.04 b	5.43 ± 0.00 b	21.37 ± 0.01 c	8.13 ± 0.01 a	2.22 ± 0.00 d	4.95 ± 0.11 b
13%	541.48 ± 0.02 b	57.68 ± 0.00 c	4.04 ± 0.00 b	31.02 ± 0.00 b	4.72 ± 0.00 d	2.45 ± 0.00 c	3.60 ± 0.01 b
18%	539.12 ± 3.13 b	57.48 ± 0.33 c	4.34 ± 0.02 c	30.92 ± 0.18 b	7.35 ± 0.01 c	2.6 ± 0.02 b	4.39 ± 1.24 b
23%	559.39 ± 1.08 a	55.21 ± 0.11 d	5.66 ± 0.01 c	34.30 ± 0.07 a	7.72 ± 0.05 b	2.74 ± 0.01 a	4.49 ± 0.37 b

Values are expressed as mean ± S.D. from triplicate determinations. The Tukey method was used for grouping information. Means in the same column that do not share a letter are significantly different at *p* < 0.05.

**Table 3:** Amino acid profile of the whole mangosteen fruit pulp including seed (MFS).

Amino Acid Profiles (g/100 g)			
Essential Amino Acids		Non-Essential Amino Acids	
Arginine	0.06	Alanine	0.10
Lysine	0.04	Aspartic acid	0.08
Histidine	0.02	Cystine	nd
Threonine	0.03	Glutamic acid	0.08
Valine	0.03	Glycine	0.05
Isoleucine	0.02	Proline	0.05
Leucine	0.05	Serine	0.03
Methionine	<0.01	Hydroxyproline	0.03
Phenylalanine	0.03	Tyrosine	0.02
Tryptophan	0.19	Alanine	0.10
Hydroxylysine	nd		

nd = not detected with LOD = 0.005; all LOD values = 0.005.

**Table 4:** Sugar profiles of fresh whole mangosteen fruit pulp including seed (MFS) and MFS-based crackers made with various MFS levels.

Sugars (%gdw)	Fresh MFS	MFS Substitution			
		0%	13%	18%	23%
Fructose	19.94 ± 0.22 a	0.17 ± 0.00 b	0.31 ± 0.00 b	0.45 ± 0.00 b	0.54 ± 0.00 b
Glucose	18.71 ± 0.21	nd	nd	nd	nd
Lactose	nd	nd	nd	nd	nd
Maltose	nd	nd	nd	nd	nd
Sucrose	33.45 ± 0.37 a	20.62 ± 0.01 b	2.05 ± 0.00 d	3.13 ±0.02 cd	3.87 ± 0.01 c
Total Sugars	72.1 ± 0.79 a	20.79 ± 0.01 b	2.37 ± 0.00 c	3.58 ± 0.02 c	4.42 ± 0.01 c

Values are expressed as mean ± S.D. from triplicate analysis. Mean values in the same row with different lowercase are significant differences (*p* < 0.05). The Tukey method was used for grouping information. nd = not detected.

**Table 5:** Mineral profiles of fresh whole mangosteen fruit pulp including seed (MFS) and MFS-based crackers made with various MFS levels.

Minerals	Units	Fresh MFS	0% MFS	13% MFS	18% MFS	23% MFS
Aluminum	mg/kgdw	nd	<1.0	<1.0	<1.0	<1.0
Boron	mg/100 gdw	0.078 ± 0.017 a	0.137 ± 0.000 c	0.016 ± 0.00 bc	0.129 ± 0.001 a	0.17 ± 0.001 b
Calcium	mg/100 gdw	63.31 ± 1.40 e	434.51 ± 0.52 d	456.41 ± 0.03 c	483.05 ± 5.59 b	523.51 ± 2.02 a
Copper	mg/100 gdw	0.33 ± 0.01 d	0.85 ± 0.00 c	0.87 ± 0.00 c	0.91 ± 0.01 b	0.95 ± 0.00 a
Iron	mg/100 gdw	0.71 ± 0.02 d	4.36 ± 0.01 a	3.70 ± 0.00 b	3.69 ± 0.04 bc	3.60 ± 0.01 c
Magnesium	mg/100 gdw	111.03 ± 2.45 e	165.18 ± 0.20 d	190.86 ± 0.01 c	212.95 ± 2.46 b	232.44 ± 0.59 a
Manganese	mg/100 gdw	0.99 ± 0.02 c	3.88 ± 0.00 a	3.24 ± 0.00 b	3.27 ± 0.04 b	3.21 ± 0.01 b
Nickel	mg/kgdw	<0.20	<0.20	<0.20	<0.20	<0.20
Phosphorous	mg/100 gdw	68.98 ± 1.52 c	862.7 ± 1.03 b	878.59 ± 0.06 ab	888.77 ± 10.28 a	884.73 ± 3.41 a
Potassium	mg/100 gdw	395.94 ± 8.73 e	1213.05 ± 1.44 d	1437.68 ± 0.11 c	1513.05 ± 17.51 b	1629.16 ± 6.27 a
Sodium	mg/100 gdw	<1.00 e	7622.2 ± 0.00 d	8545.79 ± 0.00 c	8823.73 ± 0.00 b	9201.05 ± 0.00 a
Sulfur	mg/100 gdw	60.48 ± 1.33 d	685.96 ± 0.82 b	686.69 ± 0.05 b	710.57 ± 8.22 a	652.29 ± 2.51 c
Zinc	mg/100 gdw	6.33 ± 0.14 a	5.25 ± 0.01 b	4.71 ± 0.00 c	4.89 ± 0.06 c	5.22 ± 0.02 b

Mean values ± S.D. from triplicate analysis. Mean values in the same row with different letters are significant differences (*p* < 0.05). nd = not detected.

**Table 6:** Fatty acid profiles of fresh whole mangosteen fruit pulp including seed (MFS) and MFS-based crackers made with various MFS levels.

Fatty Acids Compositions (%gdw)	Fresh MFS	Mangosteen-Based Crackers			
		0% MFS	13% MFS	18% MFS	23% MFS
Alpha-linolenic acid (C18:3, ALA, omega-3)	nd	0.16	0.21	0.21	0.23
Arachidic acid (C20:0)	0.05	0.03	0.05	0.05	0.05
Arachidonic acid (C20:4, omega-6)	nd	0.01	0.02	0.01	0.02
Behenic acid (C22:0)	nd	0.01	0.02	0.02	0.02
Butyric acid (C4:0)	nd	0.62	0.92	0.92	1.10
Capric acid (C10:0)	0.09	0.60	0.89	0.87	0.99
Caproic acid (C6:0)	nd	0.41	0.62	0.61	0.72
Caprylic acid (C8:0)	nd	0.25	0.38	0.37	0.45
Docosadienoic acid (C22:2, omega-6)	nd	nd	nd	0.02	nd
Docosahexaenoic acid (C22:6, DHA, omega-3)	nd	nd	nd	nd	nd
Eicosadienoic acid (C20:2, omega-6)	nd	nd	nd	nd	nd
Eicosapentaenoic acid (C20:5, EPA, omega-3)	nd	0.02	0.03	0.02	0.03
Eicosatrienoic acid (C20:3, omega-6)	nd	0.01	0.02	0.01	0.02
Eicosenoic acid (C20:1, omega-9)	nd	0.01	0.01	0.01	0.01
Erucic acid (C22:1, omega-9)	nd	nd	nd	nd	nd
g-Eicosatrienoic acid (C20:3, omega-3	nd	nd	nd	nd	nd
g-Linolenic acid (C18:3, omega-6)	nd	nd	nd	nd	nd
Heneicosanoic acid (C21:0)	nd	0.18	0.24	0.24	0.25
Lauric acid (C12:0)	0.05	1.01	1.49	1.46	1.60
Lignoceric acid (C24:0)	nd	0.01	0.02	0.01	0.02
Linoleic acid (C18:2, Omega-6)	1.77	0.62	0.63	0.71	0.77
Margaric acid (C17:0)	nd	0.11	0.16	0.16	0.17
Margaroleic acid (C17:1)	nd	nd	nd	nd	nd
Monounsaturated Fat	1.58	4.11	5.93	6.06	6.58
Myristic acid (C14:0)	nd	2.48	3.67	3.64	3.97
Nervonic acid (C24:1, omega-9)	nd	nd	nd	nd	nd
Oleic acid (C18:1, omega-9)	1.58	3.60	5.19	5.31	5.78
Omega-3	nd	0.18	0.24	0.24	0.26
Omega-6	1.77	0.65	0.67	0.77	0.81
Omega-9	1.58	3.61	5.20	5.33	5.80
Palmitic acid (C16:0)	0.60	6.50	9.42	9.39	10.23
Palmitoleic acid (C16:1)	nd	0.28	0.41	0.41	0.45
Pentadecanoic acid (C15:0)	nd	0.26	0.39	0.38	0.42
Pentadecenoic acid (C15:1)	nd	nd	nd	nd	nd
Polyunsaturated fat	1.77	0.83	0.91	1.01	1.07
Saturated fat	6.00	14.61	21.57	21.58	23.83
Stearic acid (C18:0)	5.16	2.07	3.21	3.39	3.78
Tetradecenenoic acid (C14:1)	nd	0.21	0.32	0.32	0.35
Trans fat	nd	0.68	1.02	1.01	1.09
trans9,12-Linolelaidic acid (C18:2trans)	nd	nd	nd	nd	nd
trans-Elaidic acid (C18:1trans)	nd	0.68	1.02	1.01	1.09
Tricosanoic acid (C23:0)	nd	0.01	0.01	0.01	0.01
Tridecanoic acid (C13:0)	nd	0.05	0.07	0.07	0.07
Unsaturated fat	3.35	4.94	6.85	7.06	7.65

nd = not detected. Before analysis, 200 g of each sample was ground up and mixed.

**Table 7:** Appearance, color, texture, and phytochemical properties of whole mangosteen fruit pulp including seed (MFS)-based crackers made with various MFS levels.

Properties	0% MFS	13% MFS	18% MFS	23% MFS
**Appearance**	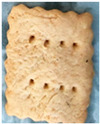	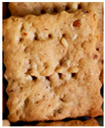	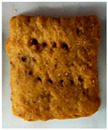	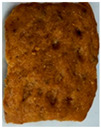
**Color**				
L*	59.65 ± 1.80 a	49.08 ± 2.90 b	49.13 ± 2.05 b	43.78 ± 0.98 c
a*	10.58 ± 0.36 b	14.50 ± 1.286 a	12.48 ± 0.27 ab	13.21 ± 1.33 ab
b*	29.72 ± 1.14 b	34.80 ± 1.52 a	30.41 ± 0.57 b	27.96 ± 0.39 b
**Texture analysis**				
Hardness (N)	17.96 ± 3.14 b	30.40 ± 0.68 a	5.74 ± 1.23 d	11.25 ± 0.68 c
Cohesiveness	0.04 ± 0.01 b	0.28 ± 0.05 a	0.07 ± 0.04 b	0.03 ± 0.02 b
**Phytochemical properties**				
TPC (mg GAE/100 g)	58.28 ± 4.49 c	107.96 ± 3.91 a	61.58 ± 1.23 c	92.88 ± 1.32 b
TFC (mg CCE/100 g)	40.84 ± 2.70 c	47.82 ± 2.66 bc	60.3 ± 2.89 b	92.2 ± 0.00 a
ARA (%)	41.78 ± 3.21 c	146.03 ± 0.45 a	49.08 ± 4.43 c	106.73 ± 3.66 b

Values are expressed as mean ± S.D. Means in the same row that do not share a letter are significantly different at *p* < 0.05. GAE = gallic equivalent. CCE = catechin equivalent.

## Data Availability

The original contributions presented in the study are included in the article; further inquiries can be directed to the corresponding author.
